# Comparison Between Electride Characteristics of Li_3_@B_40_ and Li_3_@C_60_

**DOI:** 10.3389/fchem.2021.638581

**Published:** 2021-03-15

**Authors:** Prasenjit Das, Pratim Kumar Chattaraj

**Affiliations:** ^1^ Department of Chemistry, Indian Institute of Technology Kharagpur, Kharagpur, India; ^2^ Department of Chemistry, Indian Institute of Technology Bombay, Mumbai, India

**Keywords:** endohedral encapsulation, electride, non-nuclear attractor, electron localization function basin, nonlinear optical properties properties

## Abstract

Density functional theory (DFT) based computation is performed on the endohedrally encapsulated Li_3_ cluster inside the B_40_ and C_60_ cages namely, Li_3_@B_40_ and Li_3_@C_60_. For both these systems, the Li-Li bond lengths are shorter than that in the free Li_3_ cluster. Due to confinement, the Li-Li vibrational frequencies increase in both the systems as compared to that in the free Li_3_ cluster. Thermodynamically, the formation of these two systems is spontaneous in nature as predicted by the negative values of Gibbs’ free energy changes (Δ*G*). For both the systems one non-nuclear attractor (NNA) is present on the middle of the Li_3_ cluster which is predicted and confirmed by the electron density analysis. The NNA population and the percentage localization of electron density at the NNA of the Li_3_@C_60_ system are higher than that in the Li_3_@B_40_ system. At the NNA the values of the Laplacian of electron density are negative and an electron localization function basin is present at the center of the Li_3_ cluster for localized electrons. Both systems show large values of nonlinear optical properties (NLO). Both the Li_3_ encapsulated endohedral systems behave as electrides. Electrides have low work function and hence have a great potential in catalytic activity toward the activation of small molecules (such as CO_2_, N_2_). Even some electrides have greater catalytic activity than some well-studied metal-loaded catalysts. As the systems under study behave as electrides, they have the power to show catalytic activity and can be used in catalyzing the activation of small molecules.

## Introduction

Electrons trapped inside the cavity of some interesting ionic systems behave as anions giving rise to electrides (Dye, 2003; Garcia-Borrà;s et al., 2012; Postils et al., 2015; Zhao et al., 2016; Saha et al., 2019). In recent year’s electride properties of materials have generated great attention in experiments as well as in the theoretical studies. The inception of the concept of an electride took place during the study of the solvated electrons in the solution of alkali metal systems in ammonia (Greenlee and Henne, 1946; Zurek et al., 2009). Dye et al., 1970; Dye, 1990; Dye, 1991; Dye, 1997; Dye, 2003) provided valuable studies on electride materials. The trapped electrons are not attached to any particular atom but located at the cavities and the interstices of cryptands and solid crystals, respectively (Zhao et al., 2016; Dale and Johnson, 2018). The electron density analysis confirmed the presence of confined electrons in the cavities of solid alkali metals (Marqués et al., 2009; Mei et al., 1993). Ellaboudy et al*.* (1983) synthesized the first stable organic electride, Cs^+^(18-crown-6)_2_e^–^in the crystalline form and Matsuishi et al. (2003) synthesized the first stable inorganic electride, [Ca_24_Al_28_O_64_]^4+^·(4e^–^). In both cases, the excess electrons are confined in the empty area of those crystals. This is followed by the synthesis and characterization of six temperature and air-stable electride systems (Ward et al., 1988, Ward et al.,1990a; Ward et al., 1990b; Wagner et al., 1994; Huang et al., 1997; Xie et al., 2000; Redko et al., 2005). In these systems, the cryptand ligands or crown ethers are complexed with alkali metals. Electride materials are very sensitive to temperature and air (Sun et al., 2016). So, it becomes a challenging task to generate and characterize electride materials which are stable related to air and temperature. The presence of cavity trapped loose electrons causes a lowering of the work function of electrides so that they can be used as an electron donor in chemical reactions. Moreover, the presence of loose electrons is responsible in making the electride systems very important because of their potential applications for example the emitting diodes for organic light (Yanagi et al., 2009), reversible hydrogen storage materials (Kitano et al., 2012), catalyst for the CO_2_ activation (Toda et al., 2013), splitting of N_2_ molecule (Kitano et al., 2012, 2015; Lu et al., 2016), powerful reducing agents (Buchammagari et al., 2007; Choi et al., 2014; Kim et al., 2014), and superconductivity (Miyakawa et al., 2007). The experimental identification of the position of localized trapped electrons is very difficult because of the low density of these localized electrons. So, experimentalist used indirect evidence for its experimental characterization (Singh et al., 1993; Dye, 1997). Therefore, computational studies can be helpful for the identification of electride materials. For that purpose, people used different computational tools to characterize electride materials. One can in silico characterize a material to behave as an electride and the necessary conditions for the same are, 1) presence of non-nuclear attractors (NNA) of the electron density (Dye, 2003; Lee et al., 2013; Dale et al., 2014); 2) the Laplacian of electron density (∇^2^ *ρ*) should be negative at the NNAs; 3) existence of electron localization function (ELF) basin at the NNA region; 4) high values of NLO properties. Some molecules which do not possesses confined electrons in electronic structure can show one or more of the above-mentioned properties. Thus, none of these conditions alone can be used to characterize electride systems, unambiguously. Some previous studies reported some molecules as electride material based on large NLOPs are not considered to be materials with electride properties on these days. When all of these four criteria are simultaneously satisfied, we can say that a cavity-trapped electron is present within the structure of a molecule and it constitutes a real electride material. We will analyze all of the above criteria in detail to check whether they present systems qualify to be termed as electrides. Most recently one theoretical work has shown that binuclear sandwich complexes of Be and Mg atoms bonded with isoelectronic C_5_H_5_^−^, N_5_^−^, P_5_^−^, As_5_^−^ ligands obeyed all these above-mentioned criteria to behave as electride materials (Das and Chattaraj, 2020).

After the discovery of buckminsterfullerene (C_60_) in 1985, people became interested in using its cavity for the encapsulations of metals, and gas molecules (Kroto et al., 1985). Endohedral fullerenes are very useful in biology (Cagle et al., 1996), in molecular electronics (Jaroš et al., 2019), in nuclear magnetic resonance (NMR) analysis, and in magnetic resonance imaging (Kato et al., 2003). The exterior surface of fullerenes has been used for various chemical reactions to take place (Levitt, 2013). The first experimentally synthesized endohedral fullerene is La@C_60_ (Heath et al., 1985). Experimentally, Hiroshi et al*.* reported the endohedral encapsulation of Li^+^ ion inside the C_60_ cage (Ueno et al., 2015). Experimentally Li, Ca, Pr, Y, Ba, Ce, Nd, Gd metals (Ding and Yang, 1996; Kubozono et al., 1996; Wan et al., 1998; Okada et al., 2012) and He, Ne, Ar, Kr, Xe noble gases (Saunders et al., 1993; Saunders et al., 1994; Ohtsuki et al., 1998) were kept inside the C_60_ cage. Using the “molecular surgery” approach, it is experimentally reported for the endohedral encapsulation of H_2_ (Komatsu et al., 2005; Murata et al., 2008), H_2_O (Kurotobi and Murata, 2011), HF (Krachmalnicoff et al., 2016), CH_4_ (Bloodworth et al., 2019) molecules inside fullerene. Several theoretical works have been reported for the encapsulation of different noble gases and metals inside the C_60_ cage (Andreoni and Curioni, 1996; Bühl et al., 1997; Strenalyuk and Haaland, 2008). Theoretically, Krapp and Frenking have studied the possibility of the encapsulation of noble gas dimers inside the C_60_ cage and showed the formation of noble gas-noble gas (Ng-Ng) ‘genuine’ chemical bond for Ar, Kr, and Xe, whereas weak interactions are present for He and Ne (Krapp and Frenking, 2007). Theoretically, Khatua et al. (2014a) studied the confinement of HF dimer inside the C_60_, C_70_, C_80_, and C_90_ cages. Using the *ab-initio* molecular dynamics study the movement of Ng_2_ dimer inside the C_60_ cage has been reported (Khatua et al., 2014b). Recently, endohedral encapsulation of Mg_2_ molecule inside the C_60_ cage and the bonding interactions therein have also been studied (Das et al., 2020).

Borospherene, the boron analogue of fullerene has achieved great attention to the scientist. The first reported borospherene is B_40_ having a cage-like structure (Zhai et al., 2014). After that several borospherenes such as B_28_, B_38_, B_44_, B_46_, B_29_^−^, B_37_^−^, B_38_^−^, B_39_^−^, B_44_^−^, B_39_^+^, B_40_^+^, B_41_^+^, B_42_^2+,^ and their various metal doped homologues have been reported experimentally as well as theoretically (Lv et al., 2014; Chen et al., 2015; Zhao et al., 2015; Li et al., 2016a; Li et al., 2016b; Tai and Nguyen, 2016; Tian et al., 2016; Li et al., 2017; Tai and Nguyen, 2017). Pan et al. (2018) studied the endohedral encapsulation of noble gas monomer and dimer inside the B_40_ cage and the bonding interactions between Ng-B and Ng-Ng using density functional theory (DFT). Furthermore, the endohedral encapsulation of noble gas dimer inside the cavitand of cucurbit[6]uril and octa acid has been reported (Pan et al., 2015; Chakraborty et al., 2016). Theoretically, Das and Chattaraj (2014) studied the encapsulation of alkali and alkaline earth metals inside an aza crown analogue, [(N_4_C_2_H_2_)_4_]^2-^ and the bonding interactions therein.

In this article we attempt to analyze molecules with electride property and for that purpose, we have encapsulated the Li_3_ cluster in two different cages, B_40_ and C_60_ and they are denoted as Li_3_@B_40_ and Li_3_@C_60._ We have used density functional theory (DFT) for the study of the structure, stability, and nature of bonding in these systems. We have computed the Gibbs’ free energy change for the formation of both the electride systems in gas phase as well as in toluene and benzene solvent phases. The molecular orbital analysis and the electron density analysis of both these systems have been performed. Then we have calculated and compared the NLO properties of these systems. Finally, the electride characteristics of these two systems have been analyzed and the same between them have been compared.

## Computational Details

We have used BP86-D3/def2-TZVPP method (Perdew, 1986; Becke, 1988; Weigend and Ahlrichs, 2005; Grimme et al., 2010) for geometry optimization and subsequent frequency calculations. The real harmonic frequency values indicate that these are the energy minimum structures on their respective potential energy surfaces. The Gaussian 16 program package has been used for all the computations (Frisch et al., 2016).

The atom centered density matrix propagation (ADMP) simulation has been carried out at BP86/6–31G method to know about the dynamical behavior of our systems at 300 K and 500 K temperatures and 1 atm pressure over 700 fs of time.

We have carried out the natural bond orbital (NBO) analysis to know the charge distribution on each atom. The computation for this analysis has been carried out at BP86-D3/def2-TZVPPD//BP86-D3/def2-TZVPP level of theory using NBO 3.1 version (Reed et al., 1988; Glendening et al., 1990) as implemented in Gaussian 16.

Multiwfn program package (Lu and Chen, 2012) has been used for atoms-in-molecule analysis (AIM) (Bader, 1990) of electron density. We have used BP86-D3/def2-TZVPPD//BP86-D3/def2-TZVPP method for this analysis and various bond critical points (BCP) have been generated. Both AIM and ELF basin population have been analyzed.

For the calculation of the average polarizability ($\bar{\alpha }$), first hyperpolarizability (β), and second hyperpolarizability ($\gamma_{∥}$), B3LYP/6-31+G(d)/6-31+G//BP86-D3/def2-TZVPP method has been used, where, 6-31+G(d) basis set is used for Li atoms and 6-31+G basis set is used for C and B atoms.

To compute the $\bar{\alpha }$, β and $\gamma_{∥}$ values the following equations have been used (Bishop and Norman, 2001),

$$\bar{\alpha }=\frac{1}{3}\sum_{i=x,y,z}\alpha_{ii},$$

$$\begin{array}{l} \beta ={\left(\sum_{i=x,y,z}\beta_{i}^{2}\right)}^{1/2},\\ \text{where},\beta_{i}=\frac{1}{3}\sum_{j=x,y,z}\left(\beta_{ijj}+\beta_{jij}+\beta_{jji}\right), \end{array}$$

$$\gamma_{∥}=\frac{1}{15}\sum_{i,j=x,y,z}\left(\gamma_{iijj}+\gamma_{ijij}+\gamma_{ijji}\right).$$

## Results and Discussion

### Geometries and Energetics

**FIGURE 1:**
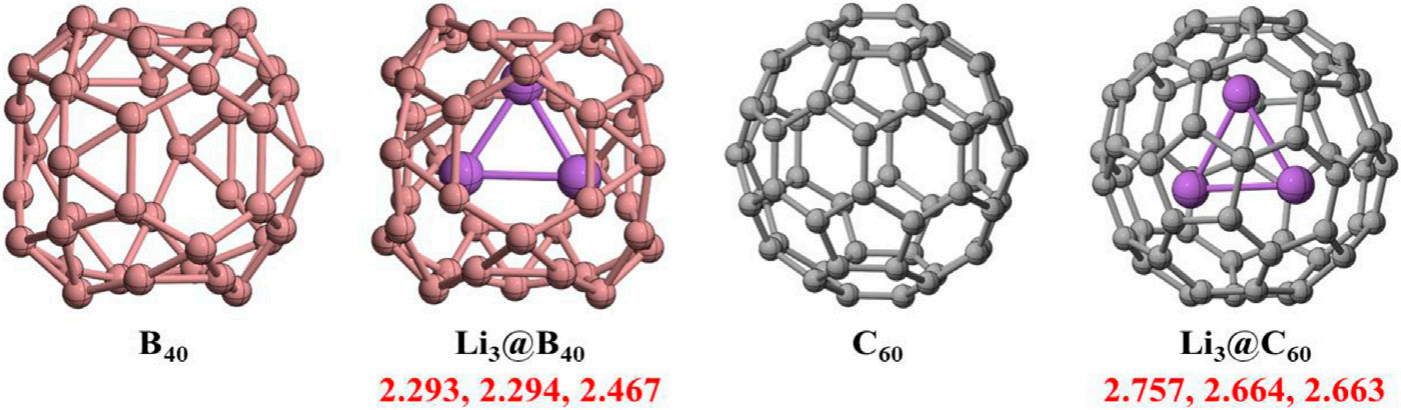
The optimized geometries of B_40_, C_60_ cages, and Li_3_@B_40_ and Li_3_@C_60_ systems at BP86-D3/def2-TZVPP method. The values in red color indicate Li-Li bond lengths (r_Li-Li_). The bond lengths are in the Å unit.

The optimized geometries of B_40_ and C_60_ cages and the Li_3_@B_40_ and Li_3_@C_60_ systems without any symmetry constraint are given in Figure 1. The B_40_ and C_60_ cages have *D*_2*d*_ and *C*_2*h*_ point groups of symmetry, respectively, and the Li_3_@B_40_ and Li_3_@C_60_ systems have *C*_1_ point group of symmetry. The Li-Li bond lengths are 2.293 Å, 2.294 Å, and 2.467 Å for the Li_3_@B_40_ system, and for the Li_3_@C_60_ system, the Li-Li bond distances are 2.757 Å, 2.663 Å, and 2.664 Å. For both the systems the Li-Li bond lengths are shorter than that in the free Li_3_ cluster. The confinement effect of B_40_ and C_60_ cages can account for this short Li-Li bond length for Li_3_@B_40_ and Li_3_@C_60_ systems, respectively. For the Li_3_@B_40_ system, the Li-Li bond lengths are shorter than that in the Li_3_@C_60_ systems. This is because of the comparatively smaller size of the B_40_ cage than that of the C_60_ cage. The numerical values of vibrational frequencies of Li-Li bonds for both Li_3_@B_40_ and Li_3_@C_60_ systems are presented in Supplementary Table S1. From the numerical values of Li-Li vibrational frequencies for both the systems, it has been found that there is an increase in the vibrational frequencies as compared to that in the free Li_3_ cluster (139.6 cm^−1^, 185 cm^−1,^ and 301.8 cm^−1^). Li_3_@B_40_ system has higher values of Li-Li vibrational frequency as compared to that of the Li_3_@C_60_ systems.

**FIGURE 2:**
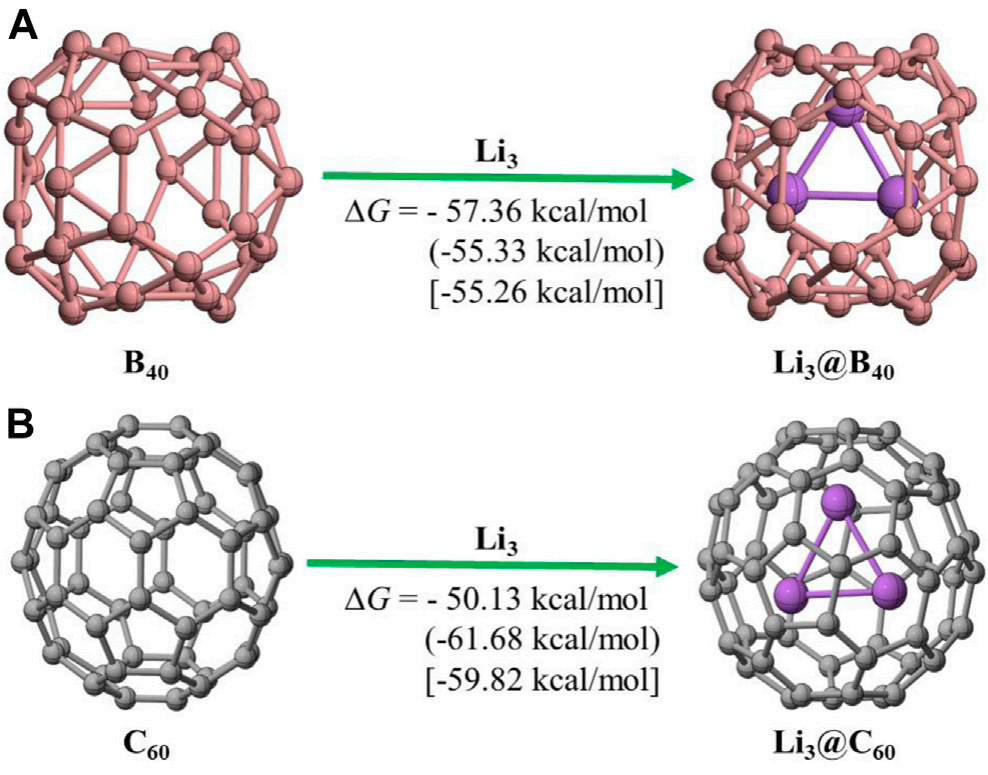
The Schematic representation of the reaction path for **(A)** Li_3_@B_40_; **(B)** Li_3_@C_60_ systems considered in the study. The values without parentheses are calculated ΔG values at gas phase. The values within parentheses and within square brackets are calculated ΔG values at toluene and benzene solvent phases, respectively.

For these endohedral encapsulation processes the Gibbs’ free energy changes (Δ*G*) in gas phase are −57.36 kcal/mol and −50.13 kcal/mol for Li_3_@B_40_ and Li_3_@C_60_ systems, respectively. For Li_3_@B_40_ system the Δ*G* values are −55.33 kcal/mol and −55.26 kcal/mol in toluene and in benzene solvents, respectively. However, for Li_3_@C_60_ system the Δ*G* values are −61.68 kcal/mol and −59.82 kcal/mol in toluene and in benzene solvents, respectively. The Δ*G* values are computed at BP86-D3/def2-TZVPP level of theory. The negative values of Δ*G* as shown in Figure 2 indicate the spontaneous formation of these endohedral systems in gas phase as well as in the solvents. So, both the hosts, B_40_ and C_60_ cages can hold and stabilize the guest Li_3_ cluster inside their cavity.

**FIGURE 3:**
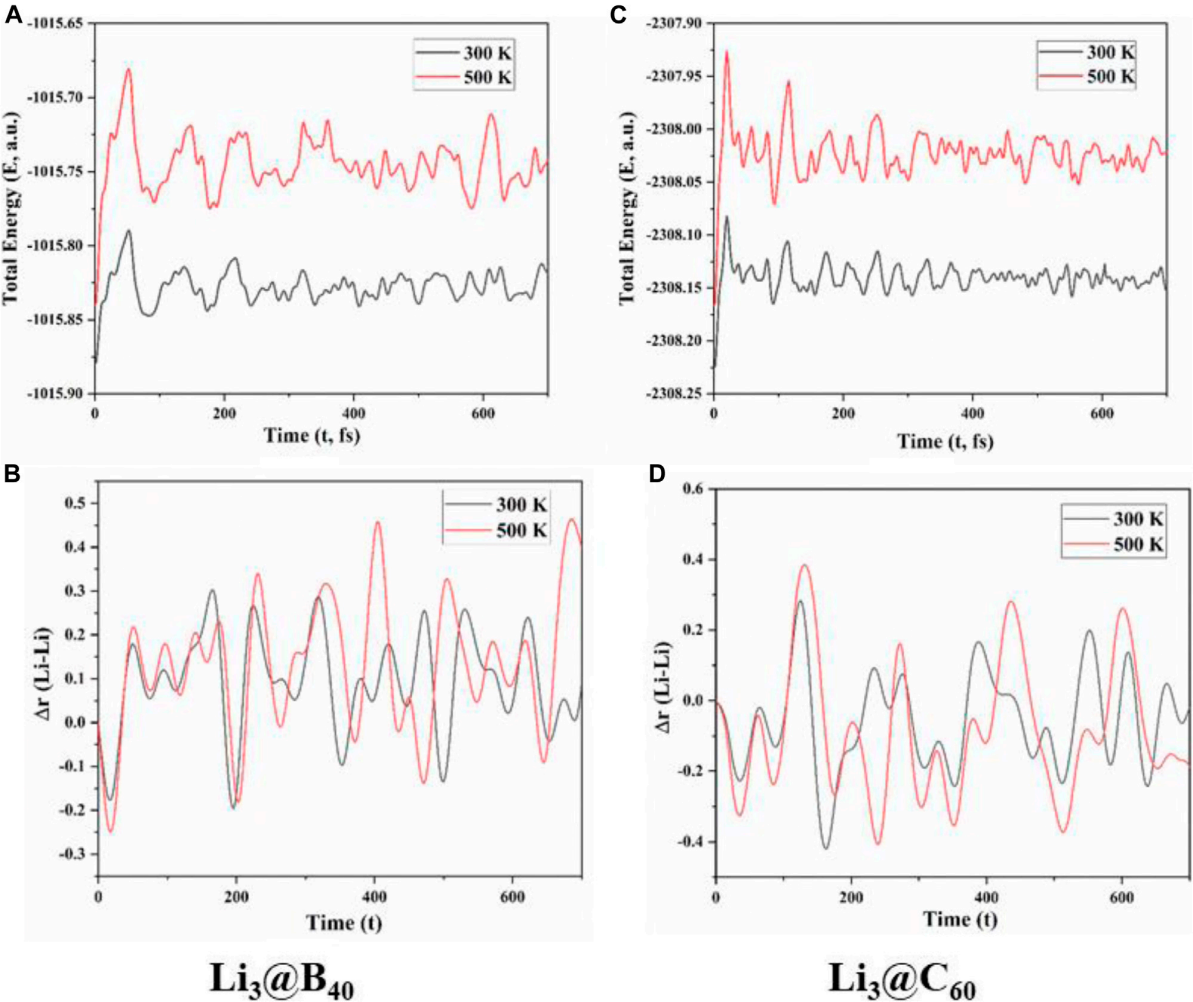
**(A)** and **(C)** Time evolution of total energy for Li_3_@B_40_ and Li_3_@C_60_ systems, respectively, **(B)** and **(D)** change in the Li-Li bond lengths with respect to the corresponding optimized value (at t = 0) against time for Li_3_@B_40_ and Li_3_@C_60_ systems, respectively.

**FIGURE 4:**
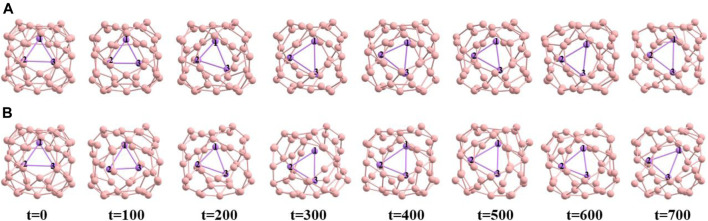
Snapshots at different time steps (time in fs) **(A)** at 300 K temperature, **(B)** at 500 K temperature of Li_3_@B_40_ system.

**FIGURE 5:**
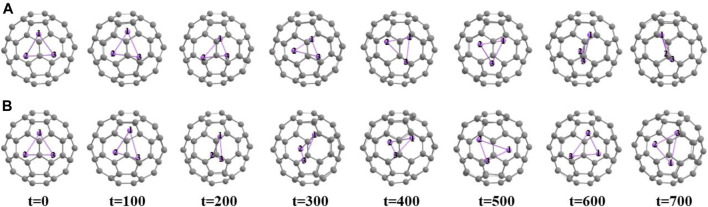
Snapshots at different time steps (time in fs) **(A)** at 300 K temperature, **(B)** at 500 K temperature of Li_3_@C_60_ system.

To know about the dynamical behavior of these systems we have carried out ADMP simulation at BP86/6-31G level of theory at both 300 K and 500 K temperature and at 1 atm pressure over 700 fs of time. We have presented the time evolution of the energy plots in Figures 3A,C for Li_3_@B_40_ and Li_3_@C_60_ systems, respectively. During structural deformation the nuclear kinetic energy of the systems increases which causes the oscillation in the time evolution of energy plots. From the time evolution plot of Li-Li bond length (Figures 3B,D for Li_3_@B_40_ and Li_3_@C_60_ systems, respectively) it is shown that the Li-Li bond lengths are fluctuating around the corresponding equilibrium values without disintegrating the systems. The different orientations of the Li_3_ cluster inside the B_40_ and C_60_ cages at these temperatures at different time steps are shown in Figures 4, 5, respectively. At both temperatures, the Li_3_ cluster is only moving inside the cages without breaking the cages. So we can say that these two systems are stable at room temperature as well as at 500 K temperature. So, the guest Li_3_ cluster can stay inside the B_40_ and C_60_ cages.

## Nature of Bonding

### Molecular Orbitals

**FIGURE 6:**
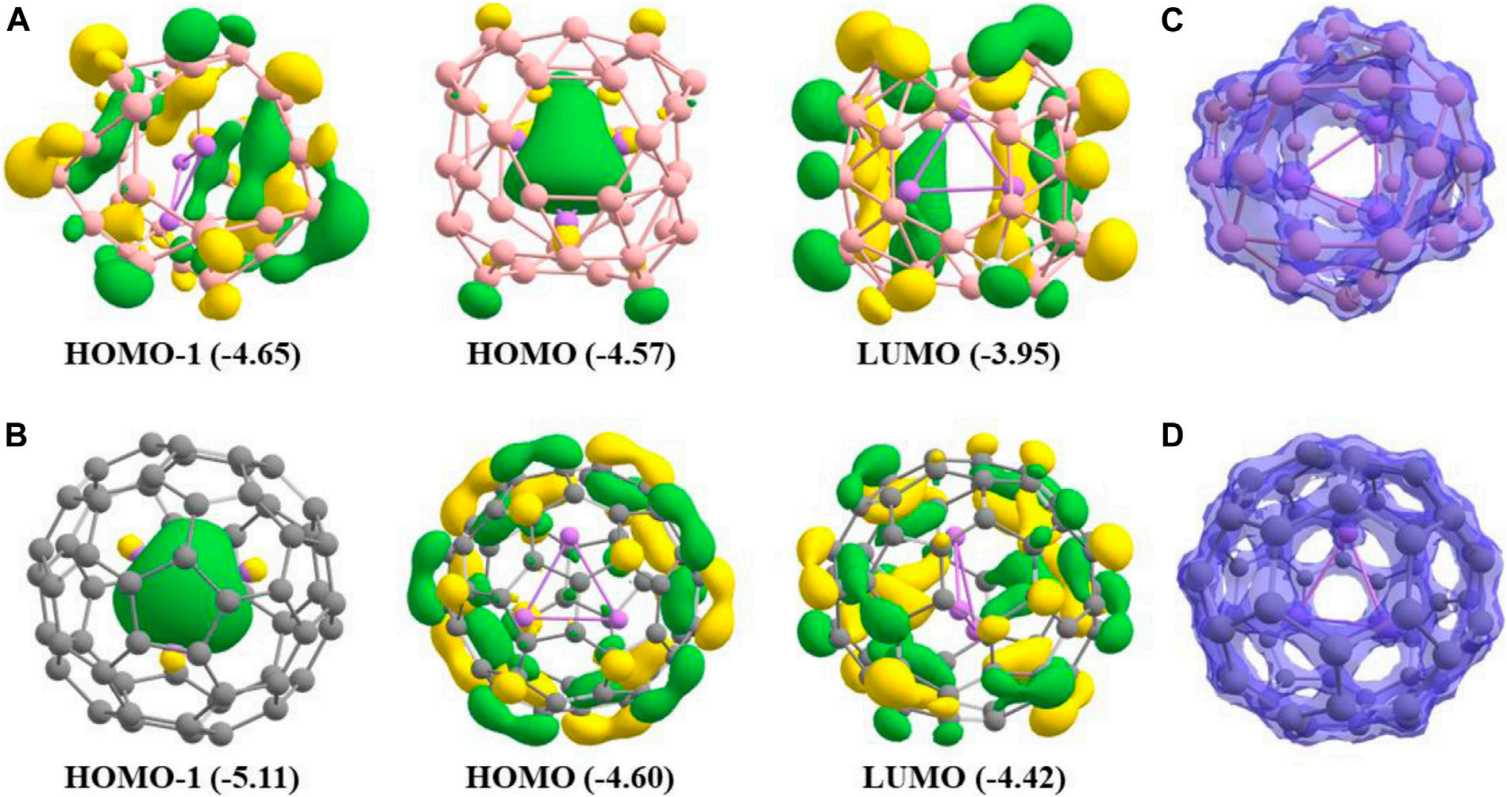
Plots of the HOMO-1, HOMO, and LUMO for **(A)** Li_3_@B_40_ system, **(B)** Li_3_@C_60_ system, **(C)** and **(D)** are spin density for Li_3_@B_40_ and Li_3_@C_60_ system, respectively. The values in the parenthesis are the energies of the corresponding orbitals in the eV unit.

We have used BP86-D3/def2-TZVPP method for molecular orbital analysis of both systems. The highest occupied molecular orbital (HOMO), HOMO-1, and lowest unoccupied molecular orbital (LUMO) for the systems are presented in Figure 6. For the Li_3_@B_40_ system, the HOMO-1 and LUMO are distributed over the B_40_ cage but there is no contribution from the Li_3_ moiety. For the Li_3_@C_60_ system, the HOMO and LUMO are π-type of orbitals and are distributed over the C_60_ cage but there is no contribution from the Li_3_ moiety. The energy differences between HOMO and LUMO are 0.62 eV and 0.18 eV for Li_3_@B_40_ and Li_3_@C_60_ systems, respectively. The spin density plots are presented in Figures 6C,D for Li_3_@B_40_ and Li_3_@C_60_ system, respectively. The spin density plots show that the total spin density is distributed over the guest Li_3_ cluster and the host B_40_ and C_60_ cages.

### Natural Bond Orbital Analysis

The charge distribution over the atoms in both the systems has been analyzed by natural bond orbital analysis. For Li_3_@C_60_ system the natural charges on Li atoms are 0.60 |*e*|, 0.60 |*e*|, and 0.55 |*e*|. While the charges on Li atoms are 0.60 |*e*|, 0.73 |*e*|, and 0.74 |*e*| for Li_3_@B_40_ system. The charges on the Li atoms are higher in case of Li_3_@B_40_ system as compared to that in the Li_3_@C_60_ system. In both the systems charge transfer takes place from the guest Li_3_ cluster to the host B_40_ and C_60_ cages. In Li_3_@B_40_ system, a greater extent of charge transfer takes place from Li_3_ to B_40_ cage as predicted by the greater positive charges on Li atoms for this system. It is reported that for La@C_82_ system 3 |*e*| transferred from the La atom to the C_82_ cage (Bethune et al., 1993). Again, in Sc_3_N@C_80_ system charge transfer occurs from Sc_3_N fragment to C_80_ cage by 6 |*e*| unit (Iiduka et al., 2005). But for F_2_@C_60_ system the charge transfer occurs in a reverse way i.e. from C_60_ cage to F_2_ molecule (Foroutan-Nejad et al., 2018).

### Atoms in Molecule Analysis

**FIGURE 7:**
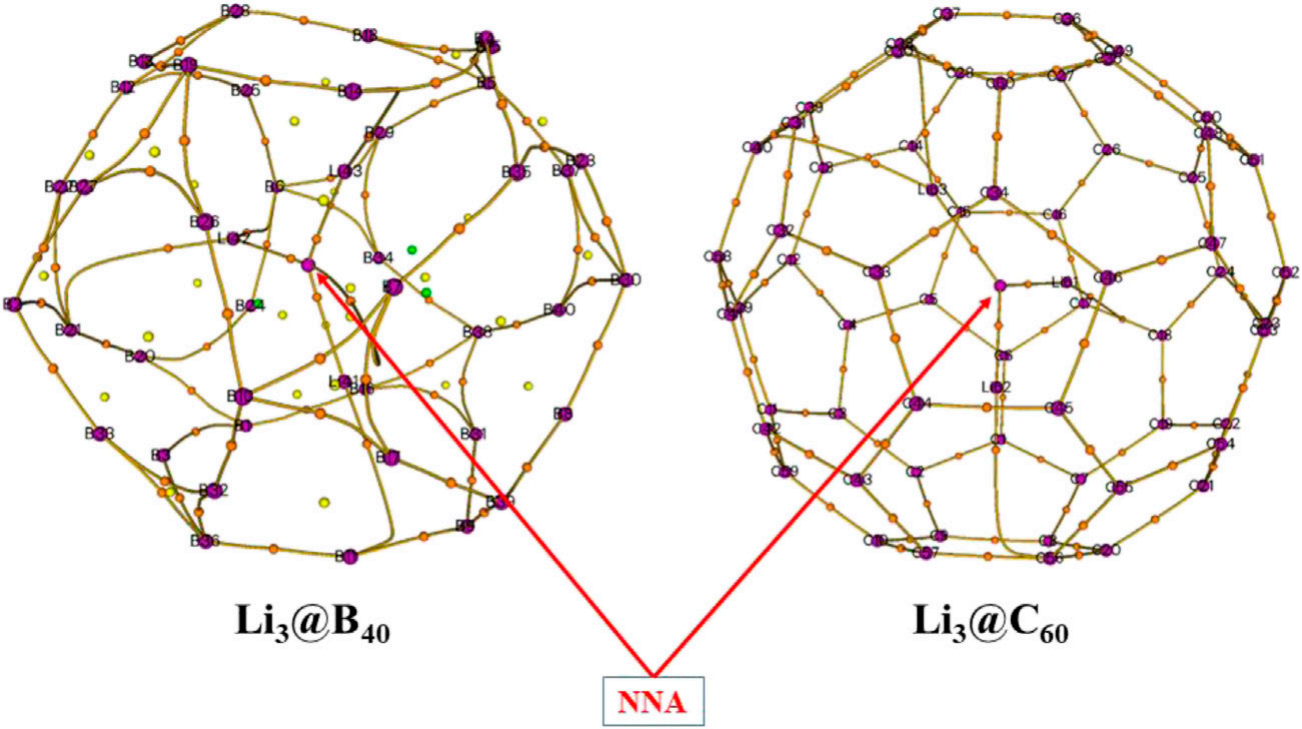
The plots of molecular graphs of Li_3_@B_40_ and Li_3_@C_60_ systems generated at BP86-D3/def2-TZVPPD//BP86-D3/def2-TZVPP level.

**FIGURE 8:**
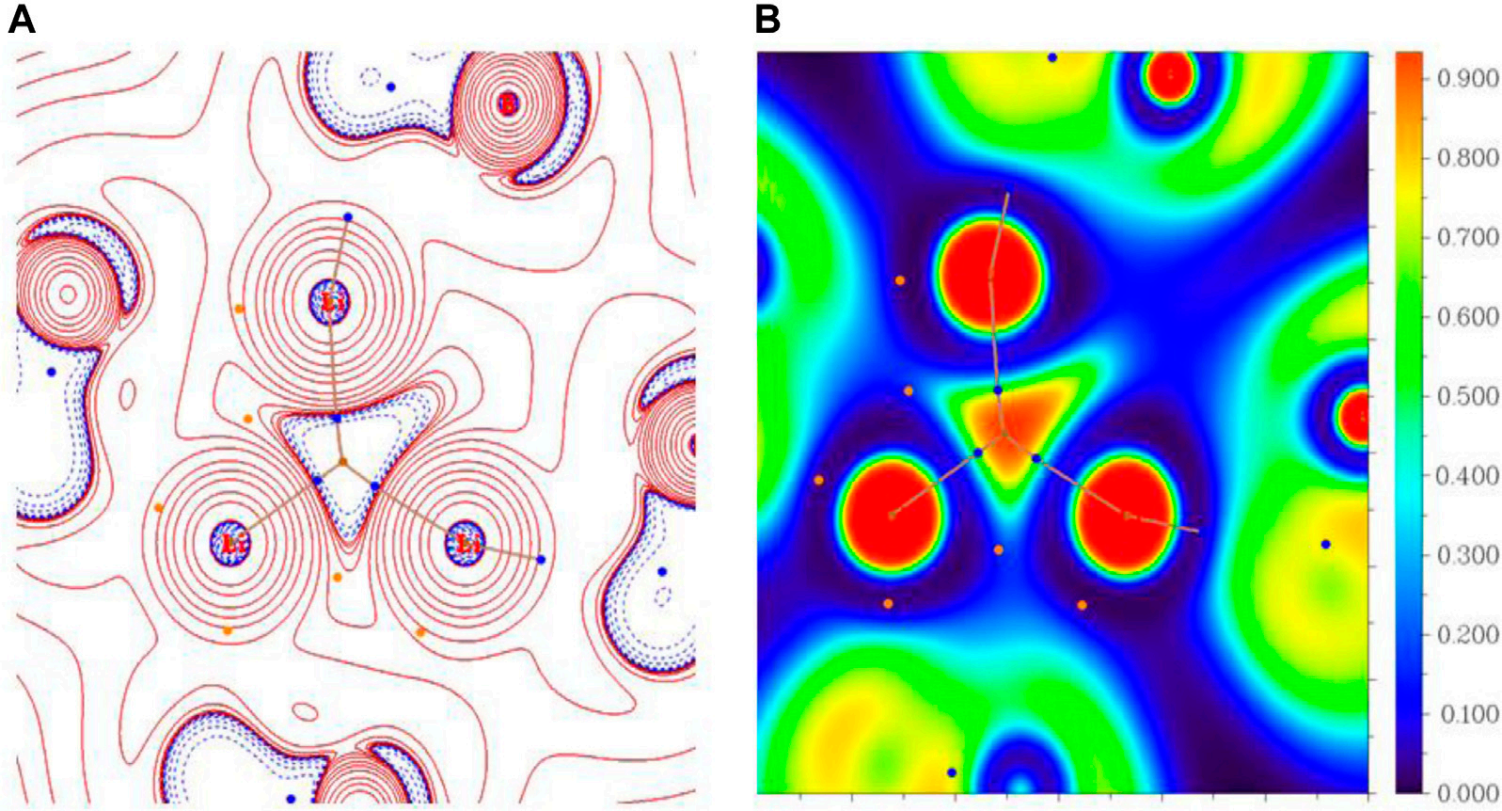
The plots of **(A)** the Laplacian of electron density [∇^2^ *ρ*(*r*)], blue dashed and red solid lines indicate ∇^2^ *ρ*(*r*) < 0 and ∇^2^ *ρ*(*r*) > 0 regions, respectively; **(B)** the electron localization function (ELF) basin of Li_3_@B_40_ and Li_3_@C_60_ systems.

The electron density descriptors of both these systems have been computed at relevant bond critical points (BCPs) and the numerical values are given in Table 1. We have also generated the corresponding molecular graphs for these systems and are presented in Figure 7. From this analysis, it is confirmed that a non-nuclear attractor (NNA) [(3, −3) type of bond critical point] is present at the center of the Li_3_ cluster for both these systems. The negative values of Laplacian of electron density [∇^2^ *ρ*(*r*_*c*_)] at both the NNAs indicate the electron localization therein. We have found that both Li_3_@B_40_ and Li_3_@C_60_ systems contained three NNA-Li bond paths which are (3,-1) type of bond critical points. The contour plots of ∇^2^ *ρ*(*r*) for both systems are presented in Figure 8A, which indicates a portion of the electron localization at the center of the Li_3_ cluster. The NNA populations are 0.17 |*e*| and 0.59 |*e*| with 12% and 46% localization of electron density for Li_3_@B_40_ and Li_3_@C_60_ systems, respectively. The population of NNA and the percentage localization of electron density at the NNA for Li_3_@C_60_ system is higher than that of the Li_3_@B_40_ system. The electron-deficient nature of boron (B) atoms may cause the lowering of percentage of localization of electron density at the NNA for Li_3_@B_40_ system as compared to Li_3_@C_60_ system, where such an effect is absent. The B_40_ cage attracts the electron density from the Li_3_ cluster more toward itself and hence decreases the electron density at the center of the Li_3_ cluster.

**TABLE 1:** Electron Density [*ρ*(r_c_)], Laplacian of Electron Density [∇^2^ *ρ*(r_c_)], Kinetic Energy Density [G(r_c_)], Potential Energy Density [V(r_c_)], Total Energy Density [H(r_c_)], Basin Population [N(pop)], Localization Index (LI), Percentage of Localization Index (% LI) at Different Critical Points (CP) of the Li_3_@B_40_ and Li_3_@C_60_ systems at BP86-D3/def2-TZVPPD//BP86-D3/def2-TZVPP level.

Systems	CP	Type	*ρ*(*r*_*c*_)	▿^2^ *ρ*(*r*_*c*_)	*G*(*r*_*c*_)	*V*(*r*_*c*_)	*H*(*r*_*c*_)	N(pop)	LI	%LI
Li_3_@B_40_	NNA	(3,−3)	0.016	−0.015	0.001	−0.006	−0.005	0.17	0.02	12
	NNA−Li	(3,−1)	0.015	−0.004	0.003	−0.008	−0.005			
Li_3_@C_60_	NNA	(3,−3)	0.018	−0.018	0.000	−0.005	−0.005	0.59	0.27	46
	NNA−Li	(3,−1)	0.015	0.004	0.005	−0.008	−0.004			

We have generated the electron localization function basin (ELF) plots for both the studied systems and are presented in Figure 8B. From the plot, it is shown that a basin is present at the center of the Li_3_ cluster for both systems. The basin population is 0.58 |*e*| with 22% localization of electron density for the Li_3_@B_40_ system. However, for the Li_3_@C_60_ system, the population of the basin is 1.01 |*e*| with 56% localization of electron density. The ELF basin population of the Li_3_@C_60_ system is higher than that of the Li_3_@B_40_ system. The lowering of the basin population for the Li_3_@B_40_ system is due to the electron-deficient nature of boron (B) atoms. From these results, it can be said that a portion of electron density is localized at the center of the Li_3_ cluster in both the systems. The higher values of NNA and ELF populations at the center of the Li_3_ cluster of Li_3_@C_60_ system as compared to the Li_3_@B_40_ system indicates a greater extent of localization of electron density in the Li_3_@C_60_ system.

### Nonlinear Optical Property

As electride materials contain loosely bound excess electrons, they showed high values of NLO properties. For this purpose, we have computed average polarizability ($\bar{\alpha }$), first hyperpolarizability (β), and second hyperpolarizability ($\gamma_{∥}$) for both the systems, and the numerical values are given in Table 2. Among both these systems Li_3_@C_60_ system shows higher values of $\bar{\alpha }$ and the Li_3_@B_40_ system shows higher values of β and$\gamma_{∥}$. We have compared the NLO values of our systems with some previously reported known electride materials, for example, M@calix (M = Li, Na; calix = calix [4]pyrrole), Li@B_10_H_14_ (Muhammad et al., 2011), and M_2_ (TCNQ) (Li et al., 2008) (M = Li, Na; TCNQ = Tetracyanoquinodimethane) and are presented in Supplementary Table S2. Our systems show comparatively higher values of $\bar{\alpha }$ but lower values of β than the systems under comparison. The numerical values of $\gamma_{∥}$ of our systems are comparable with that of the systems being compared.

**TABLE 2:** Average linear polarizability ($\bar{\alpha }$), first hyperpolarizability (*β*), and second hyperpolarizability ($\gamma_{∥}$) of Li_3_@B_40_ and Li_3_@C_60_ systems.

NLO property	Li_3_@B_40_	Li_3_@C_60_
$\bar{\alpha }$	554.2	584.7
β	129.4	79.9
$\gamma_{∥}$	3.6 × 10^5^	2.1 × 10^5^

### Electride Properties

It has been observed that in both the systems an NNA is present in the middle of the Li_3_ cluster. An ELF basin has appeared in the position where the NNA is present and the values of ∇^2^ *ρ* are negative therein. Both the systems under study exhibit high values of NLO properties. All the criteria for an electride material have been satisfied by these systems. So, Li_3_@B_40_ and Li_3_@C_60_ systems can be classified as electrides. Li_3_@C_60_ system will show better electride characteristics than the Li_3_@B_40_ system.

## Summary and Conclusion

The stability of Li_3_@B_40_ and Li_3_@C_60_ systems has been studied using density functional theory (DFT) based computations. The thermochemical results show the spontaneous formation of both the systems as predicted by the negative values of Gibbs’ free energy change (Δ*G*). Due to the confinement, the Li-Li bonds in both the systems are shorter than that in the free Li_3_ cluster and the Li-Li vibrational frequencies are increased on confinement. The Li-Li bonds are shorter in the Li_3_@B_40_ system as compared to that in the Li_3_@C_60_ system. The numerical values of Li-Li bond vibrational frequencies in the Li_3_@B_40_ system are higher than that in the Li_3_@C_60_ system. The results from the ADMP simulation showed that the systems are stable both at room temperature (300 K) and at 500 K temperature and 1 atm pressure. So, the host B_40_ and C_60_ cages can take the Li_3_ cluster inside their cavity and stabilize the cluster. The topological analysis of electron density shows the presence of an NNA at the center of the Li_3_ cluster of both these systems and a portion of electron density gets localized therein. The Laplacian of electron density is negative at the NNAs. Li_3_@C_60_ system has higher values of NNA and ELF population than that of Li_3_@B_40_ system. Our designed endohedral Li_3_@B_40_ and Li_3_@C_60_ systems behave as electride. Li_3_@C_60_ system shows better electride characteristics than Li_3_@B_40_ system. As the systems under study behave as electrides, they have the potential to show catalytic activity.

## Data Availability

The original contributions presented in the study are included in the article/Supplementary Material, further inquiries can be directed to the corresponding author.
